# Nicarbazin-Based Fertility Control for Urban Pigeon Management: A Review of Experimental, Regulatory and Operational Evidence

**DOI:** 10.3390/ani16193140

**Published:** 2026-10-07

**Authors:** Marco Pellizzari, Paolo Pietro Albonetti, Yassine Hankaz

**Affiliations:** 1 Pigeon Academy, 2518AC Den Haag, The Netherlands; yassine.hankaz@pigeonacademy.org; 2 Department of Earth, Environment and Life Sciences (DISTAV), University of Genoa, 16126 Genoa, Italy; paolopietro.albonetti@pigeonacademy.org

**Keywords:** nicarbazin, fertility control, urban pigeons, integrated pest management, population management, *Columba livia*, urban ecology

## Abstract

High urban pigeon densities can generate conflicts related to building and heritage damage, public-health concerns, nuisance, and the need for socially acceptable population-management strategies. The management of feral pigeon (*Columba livia*) populations in urban environments requires approaches that are effective, sustainable, publicly acceptable, and legally compliant. Among available non-lethal options, fertility control based on nicarbazin, originally developed in the 1950s as a poultry anticoccidial before being adapted for urban bird management, has become one of the most extensively investigated strategies. This narrative review synthesizes the scientific literature and regulatory experience concerning nicarbazin-based fertility control, including products authorized as veterinary medicinal products in Europe (Ovistop^®^, ACME DRUGS Srl, Cavriago, Italy and R12^®^, ACME Srl, Cavriago, Italy) and as an EPA-registered pesticide product in the United States (OvoControl^®^ P, Innolytics, LLC, CA, USA), covering its historical development, mechanism of action, regulatory frameworks, experimental evidence, long-term field studies, and post-marketing experience. The available evidence indicates that the effectiveness of nicarbazin is supported by multiple independent sources, including laboratory investigations, pharmacokinetic studies, long-term field evaluations, regulatory assessments, and more than two decades of operational use, although successful population management depends not only on biological efficacy but also on program implementation, monitoring, and long-term management. Based on the evidence reviewed, a Three-Pillar Framework integrating population monitoring, fertility control, and environmental and social management is proposed. Remaining evidence gaps concern the standardization of monitoring and outcome assessment, the influence of population connectivity, and the economic evaluation of long-term programs, identifying priorities for future research. Overall, nicarbazin-based fertility control emerges as a scientifically validated, reversible, and humane tool for the long-term management of urban pigeon populations within a structured, adaptive framework.

## Simple Summary

Feral pigeons are common in cities worldwide, where large populations can damage buildings, create health concerns, and cause conflict between residents and local authorities. Removing or culling pigeons rarely solves the problem for long, because new birds quickly move in to replace those that are taken away. An alternative approach uses a substance called nicarbazin, mixed into bait, which temporarily stops treated pigeons from successfully hatching eggs without harming the adult birds. This effect disappears if treatment stops, making it a reversible and humane way to slow population growth. This review brings together more than two decades of scientific studies, safety testing, and real-world experience with nicarbazin-based products used in Europe and the United States. The evidence shows that nicarbazin reliably reduces pigeon reproduction, but achieving a real drop in pigeon numbers in a city also depends on careful planning: knowing how many pigeons are present, distributing the bait consistently over several years, and combining treatment with other measures such as reducing food sources. Based on this evidence, the review proposes a simple three-part approach, monitoring, contraceptive treatment, and environmental management, to help cities manage pigeon populations effectively, humanely, and sustainably.

## 1. Introduction

Feral pigeons (*Columba livia*) are among the most widespread synanthropic birds in urban environments, where abundant anthropogenic food resources and the availability of suitable nesting and roosting sites can sustain locally high population densities [1,2]. Where pigeons become abundant, their presence may generate conflicts associated with the accumulation of droppings and consequent soiling or damage to buildings and monuments [3], public-health concerns [4], nuisance, and the management of historical and architectural heritage [3,5]. A wide range of approaches has consequently been used to manage urban pigeon conflicts [6]. However, these interventions differ substantially in their objectives, spatial scale, duration, and expected outcomes. Some are intended to reduce or stabilize pigeon populations over a defined urban area, whereas others primarily aim to prevent or reduce the presence of birds at specific sites or structures. This distinction is important when assessing and comparing the effectiveness of different management approaches.

Urban pigeon management has included population removal through trapping or culling, restriction of food resources, reproductive control, and a variety of exclusion or deterrence measures [6]. Population removal can reduce local abundance, but its longer-term effect may be limited by subsequent recruitment or immigration, particularly where the ecological conditions supporting the population remain unchanged or surrounding populations act as sources for recolonization [7,8]. Food-resource management can likewise influence population size and composition [9], whereas exclusion and deterrence measures primarily address pigeon presence at particular sites or structures [3,10]. Direct comparisons among these approaches are therefore difficult because they may target different management endpoints and operate at different spatial and temporal scales, while the supporting evidence also varies considerably in design and duration. Accordingly, evaluation of urban pigeon management requires consideration of the management objective, spatial scale, ecological context, and outcome being measured, rather than treating all control methods as directly interchangeable.

Within population-oriented management, fertility control represents a non-lethal approach aimed at reducing reproductive output rather than removing individuals from the population [11,12]. The development of nicarbazin for avian fertility control originated from reproductive effects initially observed during its use as an anticoccidial agent [13,14]. These observations provided the rationale for its subsequent development as a fertility-control agent, including the regulatory processes required for authorization. Nicarbazin-based fertility-control products were subsequently authorized under different regulatory frameworks in Europe and the United States [15,16,17]. Following authorization and operational use, field studies and longer-term monitoring programmes provided an additional layer of evidence on its application under urban conditions, particularly in feral pigeon populations [18,19,20,21]. The evidence available today therefore derives from distinct but complementary sources, ranging from the initial recognition of reproductive effects and subsequent regulatory development to post-authorization field studies and long-term population observations.

The aim of this narrative review is to critically synthesize the scientific, regulatory, and operational evidence supporting nicarbazin-based fertility control for urban feral pigeon management. The review examines the biological and pharmacological basis of nicarbazin-induced reproductive effects and their subsequent development for fertility-control purposes; describes the regulatory development and authorization of nicarbazin-based products in different jurisdictions; and assesses the field and long-term evidence generated following their operational implementation. Particular attention is given to the ecological and operational factors that may determine whether reproductive suppression translates into measurable population-level effects. Nicarbazin-based fertility control is also considered within the broader range of approaches available for urban pigeon management, taking into account differences in management objectives, spatial scale, outcome measures, and strength of supporting evidence. Finally, the review examines its role within an integrated management framework combining population monitoring, fertility control, and environmental and social measures, and identifies current evidence gaps and priorities for future research.

## 2. Materials and Methods

### 2.1. Review Design and Evidence Base

This study was conducted as a narrative review aimed at integrating scientific, regulatory, and operational evidence relevant to the use of nicarbazin-based fertility control in urban feral pigeon management. The review was developed from a continuously curated bibliographic knowledge base assembled by the authors over approximately two decades of scientific and professional activity in avian fertility control and urban pigeon management. This repository includes peer-reviewed scientific publications together with regulatory documents, institutional reports, technical monographs, and other sources considered relevant to the historical, biological, regulatory, and operational development of the subject.

Given the interdisciplinary nature of the topic, evidence was considered across several complementary domains, including the biology and population ecology of feral pigeons, nicarbazin pharmacology and reproductive effects, fertility-control applications, field and long-term population studies, safety and non-target considerations, regulatory frameworks, population monitoring, and alternative approaches to urban pigeon management. The purpose was not to perform a quantitative meta-analysis, but to critically integrate evidence originating from different study designs and source types and to interpret it in relation to the practical management of urban pigeon populations.

### 2.2. Supplementary Structured Literature Search

To update the existing bibliographic knowledge base and identify potentially relevant publications not previously included, a supplementary structured literature search was conducted in PubMed in September 2026. Three complementary search strategies were used to address: (i) nicarbazin and reproductive effects; (ii) feral pigeon population-management and control approaches; and (iii) feral pigeon monitoring, population dynamics, and integrated management.

The following search strings were applied to titles and abstracts:

Search A—Nicarbazin and reproductive effects

nicarbazin[Title/Abstract] AND (fertility[Title/Abstract] OR reproduction[Title/Abstract] OR reproductive[Title/Abstract] OR contracept*[Title/Abstract] OR hatchability[Title/Abstract] OR egg*[Title/Abstract])

Search B—Feral pigeon management approaches

(“feral pigeon*”[Title/Abstract] OR “urban pigeon*”[Title/Abstract] OR “Columba livia”[Title/Abstract]) AND (“population management”[Title/Abstract] OR “population control”[Title/Abstract] OR fertility[Title/Abstract] OR contracept*[Title/Abstract] OR culling[Title/Abstract] OR removal[Title/Abstract] OR “food restriction”[Title/Abstract] OR “food reduction”[Title/Abstract] OR deterrent*[Title/Abstract] OR exclusion[Title/Abstract] OR “pigeon loft*”[Title/Abstract])

Search C—Monitoring and population dynamics

(“feral pigeon*”[Title/Abstract] OR “urban pigeon*”[Title/Abstract] OR “Columba livia”[Title/Abstract]) AND (monitoring[Title/Abstract] OR census[Title/Abstract] OR abundance[Title/Abstract] OR “population dynamics”[Title/Abstract] OR “population estimate*”[Title/Abstract] OR “population size”[Title/Abstract] OR “integrated management”[Title/Abstract] OR “adaptive management”[Title/Abstract])

The three searches retrieved 52, 65, and 104 records, respectively, corresponding to 221 search results. After removal of records occurring in more than one search, 209 unique PubMed records were identified and screened for relevance. Comparison with the curated bibliographic knowledge base identified seven additional relevant publications not previously represented in the repository; these records were added to the knowledge base.

### 2.3. Eligibility and Evidence Selection

Records identified through the supplementary search were screened on the basis of title and abstract and compared with the existing bibliographic knowledge base to identify both previously included sources and potentially relevant additional publications. Publications were considered relevant when they contributed directly to one or more of the principal topics addressed by the review: reproductive effects or fertility-control applications of nicarbazin; feral pigeon population biology and monitoring; population-level management approaches; field implementation and long-term population outcomes; or factors affecting the interpretation and effectiveness of urban pigeon management.

Studies concerning other avian species were retained when they provided information directly relevant to the pharmacological, reproductive, exposure, or safety characteristics of nicarbazin that could inform its use for avian fertility control. Conversely, publications were excluded when the reference to pigeons or nicarbazin was incidental, when the study addressed unrelated biological or clinical questions, or when it did not provide information relevant to the objectives of the review.

Because the review addresses a heterogeneous body of evidence encompassing experimental studies, field observations, long-term monitoring, regulatory information, and management practices, no formal quantitative quality score was assigned to individual sources. Instead, evidence was interpreted according to source type, study design, duration, spatial scale, management endpoint, and relevance to the specific question under consideration.

### 2.4. Regulatory and Technical Sources

Peer-reviewed literature was complemented by primary regulatory and institutional sources where these were required to describe the authorization status, regulatory framework, or authorized use of nicarbazin-based products. These included publicly available marketing-authorisation documents for veterinary medicinal products in Europe and official U.S. Environmental Protection Agency documentation for the pesticide product OvoControl P.

Regulatory documents were used to document regulatory status and authorized uses and were distinguished from published scientific evidence. Public marketing-authorisation documents do not necessarily reproduce the experimental data or assessments contained in regulatory dossiers and were therefore not interpreted as primary evidence of efficacy, safety, or environmental effects unless such information was explicitly available in the cited public source. Technical and institutional sources were used selectively when they provided relevant historical, operational, or management information not adequately represented in the peer-reviewed literature.

## 3. Nicarbazin: Regulatory Status and Mode of Action

Nicarbazin is a synthetic equimolar complex composed of 4,4-dinitrocarbanilide (DNC) and 2-hydroxy-4,6-dimethylpyrimidine (HDP) [22]. Introduced in the 1950s as an anticoccidial agent for poultry production, it became widely used as an anticoccidial in poultry production and continues to be extensively employed today for the prevention of coccidiosis in broiler chickens and other food-producing birds [22].

Shortly after its introduction, studies conducted in laying hens revealed an unexpected effect on egg production, hatchability, and reproductive performance [8]. While this effect represented an undesirable characteristic in breeding animals, it had little practical relevance in poultry intended exclusively for meat production. Consequently, nicarbazin continued to be widely used as an anticoccidial drug for decades, while its effects on avian reproduction became progressively better understood through extensive scientific investigation and field experience [8,9,10,11,17].

The long history of use and study of nicarbazin in poultry production has generated a substantial body of information regarding its safety, metabolism, and residue characteristics [22]. Its reproductive effects subsequently stimulated investigation of nicarbazin as a fertility-control agent in birds [11,23]. The concept was based on the use of nicarbazin-induced reproductive inhibition as a humane and reversible method for controlling populations of overabundant urban birds, particularly feral pigeons. This innovative approach ultimately led to the development and regulatory authorization of dedicated nicarbazin-based fertility-control products for urban pigeon management [15,16,17].

### 3.1. Historical and Regulatory Development of Nicarbazin-Based Fertility Control

Nicarbazin-based fertility control is currently regulated under different legal frameworks depending on the jurisdiction. In the European Union, dedicated nicarbazin-based products for pigeon population control have been authorized as veterinary medicinal products at a national level.

Ovistop^®^ is authorized in Italy and R12^®^ in Belgium, while in other EU Member States nicarbazin-based veterinary medicinal products may be used, where applicable, under the cascade provisions established by Regulation (EU) 2019/6 [24,25,26]. In contrast, in the United States, nicarbazin is registered by the Environmental Protection Agency (EPA) as a pesticide product under the Federal Insecticide, Fungicide, and Rodenticide Act (FIFRA) [27]. Although these regulatory pathways differ substantially in terms of authorization procedures, labeling, distribution channels, and user requirements, all products rely on the same active substance and share the same biological mechanism of action [15,16,17].

The first authorization of a nicarbazin-based product specifically intended for avian fertility control was granted in Italy in December 2002 with Ovistop^®^, registered as a veterinary medicinal product by the Italian Ministry of Health (M.A. n. ITA-103570014) [24]. In 2007, OvoControl^®^ P was registered in the United States by the EPA as a pesticide product (EPA Reg. No. 80224-1) [27]. In 2016, R12^®^ obtained marketing authorization in Belgium as a veterinary medicinal product (M.A. n. BE-V498444) [25] (Table 1).

Since the first authorization of Ovistop^®^ in 2002, nicarbazin-based fertility control has evolved from an innovative concept into an operational management tool employed in numerous urban environments. Today, Ovistop^®^, R12^®^, and OvoControl^®^ P are used in municipalities and urban management programs across Europe and North America. Although implementation strategies vary according to local regulatory frameworks and management objectives, the continued use of these products over more than two decades reflects their continued operational use and interest in fertility control as a non-lethal approach to urban pigeon management.

### 3.2. Mechanism of Action

**Figure 1:**
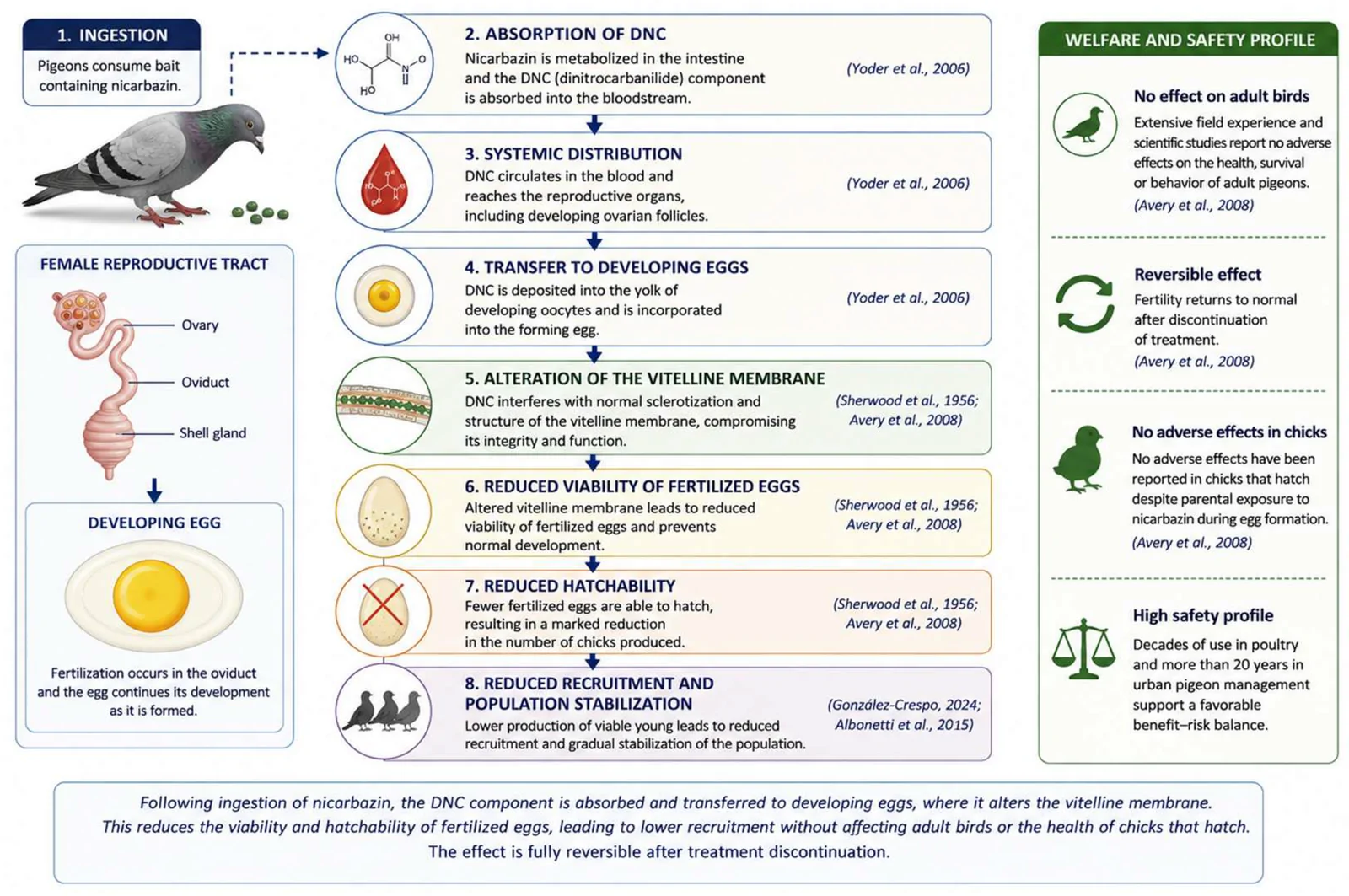
Proposed mechanism of action of nicarbazin-based fertility control in pigeons. References cited within the figure: [13,18,20,28,29,30].

Nicarbazin exerts its contraceptive activity primarily through the active moiety 4,4′-dinitrocarbanilide (DNC), which interferes with the formation and integrity of the vitelline membrane surrounding the yolk [28]. Experimental studies demonstrated a dose-dependent reduction in hatchability and embryo viability in several avian species, including waterfowl and pigeons [29,30]. Pharmacokinetic investigations documented absorption and transfer of DNC into developing eggs [31]. Field studies confirmed reduced reproductive success in feral pigeons [12,13,19]. The contraceptive effect is reversible, with reproductive function recovering following treatment cessation [19,29]. Safety and environmental studies indicate a generally favorable safety profile under the exposure conditions evaluated [32,33]. The proposed mechanism of action and the principal evidence supporting nicarbazin-based fertility control are summarized in Figure 1.

## 4. Pillar 1: Pre-Intervention Monitoring and Baseline Assessment

Initiation of a nicarbazin program without a detailed understanding of the local pigeon population is a primary cause of failure. The initial monitoring phase serves multiple critical functions:

Defining the Problem: Quantitative assessment determines whether a population exceeds the commonly cited threshold of approximately 300–400 pigeons/km^2^, above which environmental stress and management conflicts have been reported [34].

Establishing a Baseline: Accurate population estimates are essential for calculating bait requirements, positioning bait stations, and, crucially, for later measuring the program’s success. Population estimates are commonly obtained through transect counts, square sampling, and feeding-site surveys, often incorporating correction factors to account for undetected individuals and temporal variability in bird distribution [1,16,18].

Mapping the Population: Monitoring identifies key colony locations, roosting and nesting sites, primary feeding areas (both natural and human-provided), and daily movement patterns. This map informs the strategic placement of bait stations to maximize uptake by the target colonies [7,12,19].

Health and risk assessment activities can evaluate the health status of the population, identify potential zoonotic risks, and establish a baseline for future monitoring [4].

Public and Stakeholder Engagement: The data collected provide an evidence-based rationale for the program, essential for securing municipal funding and public support. It helps move the discussion from perception to measurable facts.

## 5. Pillar 2: Managed Implementation and Baiting Control

Successful application requires moving beyond simple bait distribution to active program management.

Logistics and Bait Station Management: Bait stations must be placed in sheltered, pigeon-frequented locations identified during monitoring. They require regular servicing to ensure a consistent, uncontaminated food supply and to prevent access by non-target species. Both manual bait distribution and the use of automatic timed feeders have been successfully implemented in urban fertility-control programs. The optimal method should be selected according to local environmental conditions, colony distribution, operational resources, and management objectives [19,35,36].

Addressing the “Magnet Effect”: Some urban fertility-control programs have reported a temporary increase in pigeon numbers around newly established feeding sites, likely due to the attraction of birds from surrounding areas. This phenomenon, often referred to as the “magnet effect”, is generally transient and can be mitigated through appropriate communication and management strategies [20].

Integrating Complementary Measures: A nicarbazin-based fertility-control program is most effective when implemented within an Integrated Pest Management (IPM) strategy. This approach should include public education campaigns aimed at reducing indiscriminate feeding, structural modifications to limit nesting opportunities (e.g., restricting access to cavities and ledges), and, where appropriate, the installation of physical bird deterrents on sensitive buildings. A 2003 report by Nomisma, an Italian economic research institute, on the economic and social costs associated with urban pigeons in Italy highlighted that isolated interventions rarely achieve sustainable results and that effective urban pigeon management requires a coordinated, multi-faceted approach [15].

Legal and Community Framework: Program operators must work within applicable local regulations and administrative requirements. Early engagement with animal welfare organizations, emphasizing the humane and reversible nature of fertility control, can facilitate public acceptance and reduce potential opposition. Clear public communication, including appropriate signage and informational materials, is recommended throughout the program.

## 6. Pillar 3: Annual Control and Efficacy Evaluation

Because fertility control acts by progressively reducing recruitment rather than removing existing individuals, effective programs generally require several consecutive years of implementation, often in the range of 3–5 years or longer, to achieve substantial population reductions [18,20].

Annual control cycles are essential to assess progress, adapt tactics, and justify continued investment as part of an adaptive management approach [18,20,36].

Ongoing Population Monitoring: Population counts should be repeated annually using the same methodology as the baseline survey to ensure comparability of results over time and to objectively assess population trends [18,20,36,37].

Adaptive Management: Population management should be regarded as a dynamic process rather than a fixed protocol. Data generated through annual monitoring can be used to identify factors limiting program effectiveness, including insufficient bait uptake, immigration from untreated areas, or inadequate feeder placement. Incorporating these findings into subsequent management decisions helps maximize long-term program success.

Cost–Benefit Reassessment: Annual evaluations facilitate a dynamic assessment of program costs and benefits. Changes in pigeon abundance may influence expenditures related to bait distribution, labor, cleaning operations, and damage mitigation. Monitoring these parameters over time helps determine the overall economic sustainability of the management strategy.

Long-Term Stewardship: Fertility control should be viewed as a long-term population management strategy rather than a one-time intervention. After the desired population level has been achieved, management efforts may shift from population reduction to maintenance. Continued oversight is usually required to prevent population recovery, since the environmental conditions that originally supported high pigeon densities often persist.

## 7. Case Studies and Scientific Evidence

The effectiveness of nicarbazin-based fertility control has been investigated through a combination of experimental studies, long-term field programs, and post-marketing regulatory experience. Together, these sources provide a substantial body of evidence supporting the use of nicarbazin as a non-lethal tool for the management of urban pigeon populations [18,30,37].

However, evaluating the effectiveness of fertility-control programs in free-ranging urban pigeon populations presents several methodological challenges. Unlike conventional population reduction methods, fertility control acts progressively by reducing recruitment rather than immediately removing individuals. Consequently, measurable effects on population size may require multiple breeding seasons to become evident and can be influenced by local environmental conditions, population dynamics, immigration from surrounding areas, food availability, and the quality of program implementation [11,21].

For this reason, apparent differences among studies should be interpreted with caution. In some cases, limited or inconclusive results may reflect operational constraints, insufficient treatment duration, or methodological limitations rather than a lack of biological activity of the active ingredient itself. A critical assessment of the available evidence therefore requires consideration of both the intrinsic contraceptive effect of nicarbazin and the practical factors influencing its field performance.

The following sections review the principal experimental studies, long-term urban field experiences, and regulatory evidence currently available, highlighting both the demonstrated benefits and the limitations of nicarbazin-based fertility-control programs.

### 7.1. Evidence of Efficacy and Sources of Variability

The contraceptive activity of nicarbazin has been consistently demonstrated under both experimental and field conditions. Laboratory and captive-bird studies have shown that adequate exposure to nicarbazin reduces egg hatchability and reproductive success through well-characterized mechanisms affecting egg formation and embryo development [6,8,9,10].

Nevertheless, the magnitude of population-level effects reported in urban pigeon management programs has varied among studies. While several field investigations documented substantial reductions in pigeon abundance, others reported more limited or inconclusive outcomes [11,21]. Importantly, these differences do not necessarily indicate conflicting evidence regarding the biological activity of nicarbazin. Rather, they highlight the complexity of translating contraceptive efficacy into measurable population change under real-world urban conditions.

Several factors may influence program outcomes, including treatment duration, bait palatability and consumption, availability of alternative food sources, immigration from surrounding untreated areas, population density, and the overall quality of program implementation and monitoring. Because fertility control reduces recruitment rather than directly removing adult birds, population declines are inherently gradual and may require multiple consecutive breeding seasons before becoming clearly detectable.

### 7.2. Experimental Evidence

The biological basis of nicarbazin-based fertility control is supported by several decades of experimental research. Early investigations demonstrated that nicarbazin interferes with avian reproduction by reducing egg hatchability and embryo viability, effects that were initially observed in domestic poultry and later confirmed in other bird species [13].

Subsequent studies clarified the underlying mechanism of action. Nicarbazin acts primarily through its active component 4,4′-dinitrocarbanilide (DNC), which disrupts the formation and integrity of the vitelline membrane surrounding the yolk. This alteration compromises normal egg development and significantly reduces reproductive success without inducing permanent infertility [28]. Pharmacokinetic investigations further demonstrated the transfer of DNC from treated females into developing eggs, establishing a direct link between product consumption and reproductive effects [29].

The contraceptive effect has also been demonstrated under controlled experimental conditions in pigeons. Martelli et al. [23] reported a marked reduction in reproductive performance in captive feral pigeons receiving nicarbazin-treated feed. Similarly, Avery et al. [30] demonstrated a significant reduction in hatchability and chick production in treated pigeon pairs, confirming that the contraceptive effect observed in poultry is equally applicable to feral pigeons. These findings provided the experimental foundation for the subsequent development of fertility-control programs in free-ranging urban pigeon populations.

Importantly, experimental studies consistently indicate that the effectiveness of treatment depends on adequate product intake. Factors such as dose, duration of exposure, and food consumption influence the magnitude of the reproductive response, highlighting the importance of ensuring sufficient uptake under field conditions [11]. Nevertheless, the biological capacity of nicarbazin to reduce fertility has been repeatedly demonstrated under controlled conditions and is considered one of the most thoroughly investigated aspects of its use in avian population management.

### 7.3. Long-Term Urban Field Studies

Long-term field studies provide the most relevant evidence for evaluating the practical effectiveness of nicarbazin-based fertility control under real urban conditions. Unlike laboratory experiments, these investigations must account for the complexity of free-ranging pigeon populations, including immigration, alternative food sources, seasonal fluctuations, and variations in treatment uptake. Consequently, they represent a critical bridge between experimental efficacy and real-world population management.

One of the earliest large-scale evaluations was conducted in the city of Genoa, Italy. Albonetti et al. [20] retrospectively assessed the use of Ovistop^®^ in several urban colonies and reported a substantial reduction in pigeon abundance over time. The study demonstrated that fertility control could contribute to both population stabilization and progressive reduction when treatment was maintained consistently and integrated into a structured management program. Importantly, the authors emphasized that population responses became more evident over successive years of implementation, supporting the concept that fertility control is inherently a long-term strategy rather than a rapid population-reduction tool.

Additional evidence has recently been provided by a long-term monitoring study conducted in Genoa between 2010 and 2017. Albonetti et al. [37] documented a marked reduction in pigeon abundance within the Higher-Central District, with population decreases approaching 70% in some monitored areas. The study highlighted the importance of systematic monitoring, consistent bait distribution, and adaptive management practices, demonstrating that substantial and sustained reductions can be achieved without resorting to lethal control methods.

Comparable results have been reported in Barcelona, Spain. González-Crespo and Lavín [19] evaluated a fertility-control program based on nicarbazin administered through automatic feeders over a three-year period. The study documented a progressive reduction in pigeon abundance, reaching an overall decline of 55.26% by the end of the monitoring period, together with a significant reduction in juvenile recruitment. The authors concluded that fertility control represented an effective and animal welfare-friendly strategy for the management of conflictive urban pigeon colonies.

Interestingly, the Barcelona experience provides a useful example of how study duration can influence the interpretation of program outcomes. Senar et al. [21] did not detect a significant reduction in pigeon abundance after approximately one year of treatment, whereas González-Crespo and Lavín [19] documented a progressive decline reaching 55.26% after three years of continuous implementation. Together, these studies suggest that short-term evaluations may underestimate the cumulative effects of fertility-control programs and reinforce the importance of multi-year monitoring.

Taken together, these field studies indicate that nicarbazin-based fertility control can produce substantial reductions in urban pigeon populations when applied consistently over multiple years. Although the magnitude and speed of population decline may vary according to local ecological and management conditions, the overall evidence suggests that long-term program implementation is one of the principal determinants of success.

### 7.4. Regulatory Validation and Pharmacovigilance

In addition to experimental and field evidence, the regulatory history of nicarbazin-based fertility-control products provides an independent source of information regarding their efficacy, safety, and environmental compatibility under practical conditions of use.

In Europe, Ovistop^®^ was first authorized in Italy in 2002 as a veterinary medicinal product for the reproductive control of feral pigeons [24]. Subsequently, R12^®^ received marketing authorization in Belgium in 2016 [25]. The granting of marketing authorizations for veterinary medicinal products requires the submission of regulatory dossiers containing documentation addressing product quality, safety and efficacy, including, where applicable, environmental risk assessment. These dossiers are evaluated by the competent regulatory authorities as part of the marketing authorization procedure [16].

Outside Europe, OvoControl^®^ P was registered in the United States in 2007 by the Environmental Protection Agency (EPA) as a pesticide product under the Federal Insecticide, Fungicide, and Rodenticide Act (FIFRA) [27]. 

Regulatory oversight does not end with product authorization. Within the European Union, veterinary medicinal products remain subject to continuous post-marketing surveillance through pharmacovigilance systems established to monitor adverse events, environmental concerns, and the overall benefit–risk balance throughout the product life cycle. Regulatory authorities retain the power to impose restrictions, suspend authorizations, or withdraw products from the market should significant concerns regarding safety or efficacy emerge [26].

The regulatory experience accumulated with nicarbazin-based fertility-control products is therefore noteworthy. Ovistop^®^ received an unlimited renewal of its marketing authorization in 2007 [17], while the marketing authorization for R12^®^ was renewed in 2022 [38]. 

An additional aspect that should be considered when interpreting the available evidence is the distinction between the scientific literature and the broader operational experience accumulated over time. While the number of peer-reviewed studies specifically evaluating fertility control in urban pigeons remains relatively limited, nicarbazin itself is among the most extensively studied and widely used compounds in avian medicine. Moreover, nicarbazin-based fertility-control products have been employed for more than two decades in numerous urban-management programs operating under different regulatory frameworks and subject to ongoing regulatory oversight. Consequently, the practical experience accumulated through routine implementation and post-marketing surveillance is substantially broader than the evidence currently documented in scientific literature alone. The principal scientific, regulatory, and operational evidence discussed in this review is summarized in Table 2.

## 8. Discussion

The literature reviewed in this paper indicates that nicarbazin-based fertility control represents one of the most extensively investigated approaches currently available for the management of urban pigeon populations. Evidence supporting its use derives from multiple and independent sources, including experimental studies, long-term field investigations, regulatory evaluations, and post-marketing surveillance. Collectively, these data provide a broader and more robust evidence base than is available for many other interventions commonly employed in urban bird management.

### 8.1. Strength of the Available Evidence

One of the most notable findings emerging from this review is the breadth and diversity of the evidence supporting the use of nicarbazin-based fertility control for urban pigeon management. 

Experimental studies have consistently demonstrated the biological activity of nicarbazin and clarified its mechanism of action, including its effects on egg hatchability, embryo development, and reproductive success [6,8,9,10]. These findings have been complemented by pharmacokinetic investigations documenting the transfer of DNC into developing eggs and supporting the causal relationship between product consumption and contraceptive effect [9,10].

Evidence generated under controlled conditions has subsequently been supported by field studies conducted in free-ranging urban pigeon populations. Long-term investigations in cities such as Genoa and Barcelona have demonstrated that fertility-control programs can produce substantial reductions in population size when implemented consistently and monitored over multiple years [19,20,37].

A further strength of the available evidence derives from the regulatory history of nicarbazin-based products. Ovistop^®^, R12^®^, and OvoControl^®^ P have been subject to authorization or registration procedures under their respective regulatory frameworks [15,16,17].

Taken together, these complementary lines of evidence provide a comprehensive body of knowledge for an urban wildlife management tool. While no single study can definitively resolve all questions regarding effectiveness under every possible operational scenario, the convergence of experimental, field, regulatory, and post-marketing evidence supports the scientific basis of nicarbazin-based fertility control as a management option for urban pigeon populations [6,7,12,13,15,16,17,19,39,40].

### 8.2. Why Results Differ Among Studies

Although the overall body of evidence supports the effectiveness of nicarbazin-based fertility control, considerable variation exists in the magnitude and timing of the population-level responses reported among different studies. This variability has occasionally been interpreted as conflicting evidence regarding the effectiveness of fertility control. However, a closer examination of the available literature suggests that many of these differences are more likely attributable to ecological and operational factors than to uncertainty regarding the biological activity of nicarbazin itself.

Unlike conventional population-control methods that directly remove individuals from a population, fertility control acts indirectly by reducing recruitment. As a result, measurable changes in population size may require several breeding cycles before becoming fully apparent. The duration of monitoring therefore represents a critical factor when evaluating program outcomes.

The experience from Barcelona provides a particularly informative example. Senar et al. [21] reported no significant reduction in pigeon abundance after approximately one year of treatment. In contrast, González-Crespo and Lavín [19], evaluating the same urban context over a three-year period, documented a progressive population decline exceeding 50%. Considered together, these studies suggest that short-term assessments may underestimate the cumulative effects of fertility-control programs and highlight the importance of long-term monitoring when interpreting field results.

Several additional factors may influence program performance. These include the proportion of the target population consuming the treated bait, the availability of alternative food sources, immigration from surrounding untreated areas, population density, and the consistency of bait distribution. Giunchi et al. [11] emphasized that even highly effective fertility-control agents may produce limited population-level effects if treatment coverage is incomplete or if ecological conditions facilitate rapid population recovery.

Consequently, studies reporting limited effects should be interpreted within their operational context. Current evidence suggests that variability among programs is more often related to ecological and management factors than to uncertainty regarding the contraceptive action of nicarbazin itself. Long-term, well-monitored programs generally provide the most reliable assessment of treatment effectiveness.

### 8.3. From Fertility Control to Population Management

The distinction between biological efficacy and operational effectiveness has important implications for urban pigeon management. While the contraceptive activity of nicarbazin has been consistently demonstrated under both experimental and field conditions, the success of a fertility-control program ultimately depends on how the intervention is integrated into a broader management framework.

In this context, the principles of Integrated Pest Management (IPM) provide a useful framework for program design and implementation [15]. Similar concepts have been proposed more broadly in wildlife management, where fertility control is considered most effective when integrated with monitoring, stakeholder engagement, and complementary management measures rather than implemented as a stand-alone intervention [6,12,36].

Within such a framework, fertility control should be regarded not as an isolated intervention but as one component of a comprehensive population-management strategy.

### 8.4. Comparison with Other Urban Pigeon Management Approaches

Alternative approaches to urban pigeon management operate at different spatial scales and pursue different management objectives. Area-based population management approaches include removal or culling, food-resource management, and, where applicable, reproductive control through egg or nest management. In contrast, site-specific approaches such as exclusion systems, nets, spikes, and behavioural deterrents are primarily intended to reduce pigeon presence at particular buildings or sensitive locations [23,30,31,32,33,34,36].

Traditional trapping and culling programs, in particular, have often produced only temporary reductions in pigeon abundance because removed individuals may be rapidly replaced through recruitment and immigration. Similar limitations have been documented in several European urban-management experiences, including Barcelona, where previous studies identified juvenile replacement and increased reproductive output as major factors reducing the long-term effectiveness of removal-based strategies [39].

Falconry and physical or behavioural deterrents primarily address bird presence at specific sites rather than population recruitment at the urban scale. Their outcomes should therefore not be directly compared with those of area-based population-management interventions using changes in overall population abundance as the common endpoint [3,6,10]. Food-resource management represents another area-based approach, aiming to reduce environmental carrying capacity rather than directly removing birds or suppressing reproduction. Reductions in anthropogenic food availability have been associated with decreases in feral pigeon abundance, although outcomes depend on the extent and persistence of food-resource control [9].

In contrast, fertility-control approaches act directly on population recruitment while providing a reversible contraceptive effect and a favorable safety profile for target animals and the environment, supported by both experimental studies and independent regulatory assessments [28,29,30,33].

### 8.5. Remaining Knowledge Gaps

Despite the substantial body of evidence supporting nicarbazin-based fertility control, several important knowledge gaps remain and should be addressed by future research.

One limitation of the current literature is the lack of standardized methodologies for population monitoring and outcome assessment. Differences in census techniques, study duration, treatment protocols, and reporting criteria make direct comparisons among studies difficult and may contribute to the variability of published results. The development of standardized monitoring protocols would facilitate comparisons among cities and improve the interpretation of long-term population trends.

A second area requiring further investigation concerns the influence of immigration and metapopulation dynamics on program outcomes. Urban pigeon populations are rarely isolated, and movements between treated and untreated areas may significantly affect the apparent effectiveness of fertility-control programs. Future studies should therefore consider broader spatial scales and incorporate population-connectivity analyses where possible.

Additional research is also needed to evaluate the economic aspects of fertility-control programs. Although reductions in cleaning costs, structural damage, and management expenditures are frequently cited as potential benefits, relatively few studies have quantified these outcomes using standardized economic methodologies [15].

Finally, the literature would benefit from the development of evidence-based operational guidelines defining best practices for the implementation of fertility-control programs. The evidence reviewed in this paper suggests that factors such as monitoring intensity, bait-delivery strategies, treatment duration, stakeholder engagement, and complementary management measures may substantially influence program success. Establishing standardized frameworks for program implementation would improve both operational consistency and the comparability of future studies.

Addressing these knowledge gaps will not only strengthen the scientific basis of fertility-control programs but also support the development of more effective, transparent, and evidence-based strategies for the long-term management of urban pigeon populations.

## 9. Conclusions

The evidence reviewed in this paper indicates that nicarbazin, marketed as Ovistop^®^, R12^®^, and OvoControl^®^ P, represents a scientifically validated, reversible, and ethically acceptable tool for the management of urban feral pigeon populations. Its effectiveness is supported by experimental studies, long-term field investigations, regulatory evaluation, and post-marketing surveillance, providing a uniquely broad evidence base among currently available urban pigeon-management approaches.

At the same time, the literature consistently shows that biological efficacy alone does not guarantee successful population management. The long-term outcomes of fertility-control programs depend on their integration within a structured management framework combining scientific population monitoring, sustained fertility-control interventions, and complementary environmental and stakeholder-management measures.

The present review therefore supports a shift from viewing fertility control as a simple contraceptive treatment to recognizing it as a comprehensive population-management strategy. The technical potential of nicarbazin can only be fully realized when fertility-control programs are designed, implemented, and evaluated within a structured framework based on the three complementary pillars identified in this review: scientific monitoring, fertility control, and environmental and social management.

By adopting this approach, veterinarians, municipalities, and wildlife-management professionals can develop evidence-based programs capable of achieving sustainable population regulation while reducing human–wildlife conflicts and protecting public health, infrastructure, and cultural heritage in a humane and socially acceptable manner [40].

Remaining limitations include the lack of standardized monitoring and outcome-assessment methods, uncertainties regarding immigration and population connectivity, and limited economic evaluations of fertility-control programs. Future research should therefore focus on standardized monitoring protocols, broader spatial analyses of population dynamics, economic assessment, and evidence-based operational guidelines to improve the comparability and long-term evaluation of management programs.

## Figures and Tables

**Table 1:** Historical Development of Nicarbazin-Based Fertility Control. Sources: [15,16,17].

Product	Country	Year	Regulatory Framework
Ovistop^®^	Italy	2002	Veterinary medicinal product
OvoControl^®^ P	USA	2007	EPA-registered pesticide product
R12^®^	Belgium	2016	Veterinary medicinal product

**Table 2:** Key Scientific, Regulatory, and Operational Evidence Supporting Nicarbazin-Based Fertility Control in Urban Pigeons. The table summarizes the principal sources of evidence discussed in this review, including mechanistic studies, pharmacokinetic investigations, field evaluations, regulatory authorizations, and post-marketing experience.

Evidence Category	Reference/Program	Location	Main Contribution
Mechanism of action	Sherwood et al. [13]	USA	First description of the reproductive effects of nicarbazin and reduced hatchability.
Pharmacokinetics and reproductive effects	Yoder et al. [28,29]	USA	Characterization of DNC transfer to eggs and relationship between exposure and reproductive inhibition.
Safety and reversibility	Avery et al. [30]	USA	Demonstrated reversibility of the contraceptive effect and absence of adverse effects in adult pigeons and chicks that hatched despite exposure.
Field implementation	Albonetti et al. [20]	Genoa, Italy	Demonstrated population reduction under long-term operational conditions.
Short-term municipal program	Senar et al. [21]	Barcelona, Spain	Highlighted the limitations of one-year evaluations.
Long-term municipal program	González-Crespo & Lavín [19]	Barcelona, Spain	Demonstrated substantial population reduction after three consecutive years of treatment.
Multi-city implementation	González-Crespo [18]	24 municipalities in Catalonia, Spain	Confirmed effectiveness across multiple municipalities and management scenarios.
Operational management	Albonetti et al. [36]	Genoa, Italy	Proposed standardized implementation criteria and management recommendations.
Regulatory authorization	Ovistop^®^ (2002–present)	Italy	Veterinary medicinal product authorized for urban pigeon fertility control.
Regulatory authorization	OvoControl^®^ P (2007–present)	USA	EPA-registered fertility-control product used in urban bird management programs.
Regulatory authorization	R12^®^ (2016–present)	Belgium	Veterinary medicinal product authorized for urban pigeon fertility control.
Post-marketing surveillance	Ovistop^®^, OvoControl^®^ P, R12^®^	Multiple jurisdictions	More than two decades of operational use under regulatory oversight and post-marketing monitoring.

## Data Availability

No new data were created in this review. All data discussed are available in the cited references.

## Abbreviations

The following abbreviations are used in this manuscript:

Abbreviation	Definition
DNC	4,4′-Dinitrocarbanilide
HDP	2-Hydroxy-4,6-dimethylpyrimidine
EPA	United States Environmental Protection Agency
EFSA	European Food Safety Authority
IPM	Integrated Pest Management
